# Using Privacy Respecting Sound Analysis to Improve Bluetooth Based Proximity Detection for COVID-19 Exposure Tracing and Social Distancing

**DOI:** 10.3390/s21165604

**Published:** 2021-08-20

**Authors:** Gernot Bahle, Vitor Fortes Rey, Sizhen Bian, Hymalai Bello, Paul Lukowicz

**Affiliations:** 1 German Research Center for Artificial Intelligence, Trippstadter Str. 122, 67663 Kaiserslautern, Germany; vitor.fortes@dfki.de (V.F.R.); sizhen.bian@dfki.de (S.B.); Hymalai_Yenireth.Bello_Valera@dfki.de (H.B.); paul.lukowicz@dfki.de (P.L.); 2 Informatics Department, University of Kaiserslautern, Erwin-Schroedinger-Strasse 1, 67663 Kaiserslautern, Germany

**Keywords:** COVID-19, proximity detection, Bluetooth, sound similarity

## Abstract

We propose to use ambient sound as a privacy-aware source of information for COVID-19-related social distance monitoring and contact tracing. The aim is to complement currently dominant Bluetooth Low Energy Received Signal Strength Indicator (BLE RSSI) approaches. These often struggle with the complexity of Radio Frequency (RF) signal attenuation, which is strongly influenced by specific surrounding characteristics. This in turn renders the relationship between signal strength and the distance between transmitter and receiver highly non-deterministic. We analyze spatio-temporal variations in what we call “ambient sound fingerprints”. We leverage the fact that ambient sound received by a mobile device is a superposition of sounds from sources at many different locations in the environment. Such a superposition is determined by the relative position of those sources with respect to the receiver. We present a method for using the above general idea to classify proximity between pairs of users based on Kullback–Leibler distance between sound intensity histograms. The method is based on intensity analysis only, and does not require the collection of any privacy sensitive signals. Further, we show how this information can be fused with BLE RSSI features using adaptive weighted voting. We also take into account that sound is not available in all windows. Our approach is evaluated in elaborate experiments in real-world settings. The results show that both Bluetooth and sound can be used to differentiate users within and out of critical distance (1.5 m) with high accuracies of 77% and 80% respectively. Their fusion, however, improves this to 86%, making evident the merit of augmenting BLE RSSI with sound. We conclude by discussing strengths and limitations of our approach and highlighting directions for future work.

## 1. Introduction

Social distancing remains one of the most effective measures for containing the COVID-19 pandemic (and many other diseases that are spread through droplets). It is defined as keeping at least 1.5 to 2 m distance between people. In terms of tracking and interrupting the infection chains, a key piece of information is being able to reliably identify who has been in close proximity (below the social distancing range) with a potentially infectious individual. Given the fact that in the industrialized world, the vast majority of the population constantly have their smartphones with them, using smartphone sensing to monitor social distancing is an obvious idea. Due to this, a wide range of social distancing monitoring apps have emerged throughout the past year, with nearly every country having at least one official corona tracing/warning app. In terms of sensing technology, virtually all those apps rely on Bluetooth Low Energy (BLE) which is a standard short-range communication technology in today’s mobile devices [1]. The general idea is for each device to alternate between broadcasting short messages and listening for messages broadcast by others and then using the Received Signal Strength Indicator (RSSI) of the received messages as an indication of the distance to the owner of the respective device. The main advantage of that approach is the fact that it can be fairly easily implemented on most standard mobile phones and that sensing can be combined with the exchange of information needed to build a contact tracing functionality on top of the derived proximity information. In fact, in iOS and Android there is now even direct operating system support for this type of contact tracing, which includes not only proximity detection but also the entire communication and contract tracing infrastructure with an elaborate privacy protection policy. On the other hand using BLE RSSI for social distancing monitoring and contact tracing is not without problems:

1. In terms of infection risk, proximity becomes irrelevant if there is a physical barrier in between. Such barriers are often used in supermarkets, restaurants and offices. People may also often sit next to each other distance-wise but be separated by a thin wall. Often such barriers are more or less transparent to 2.4 GHz signals so that BLE RSSI-based contact tracing systems mistakenly assume an infection risk.
2. While under ideal conditions (i.e., in empty space) distance can be estimated exactly using signal attenuation, in the real world, RF signal strength is determined by a combination of factors such as absorption, reflection and diffraction. In particular, the human body is highly absorbent at 2.4 GHz, which means that for the same distance between two people facing each other, the RSSI value will be very different depending on whether phones are in front or back pockets. In general, the phone orientation, placement and other factors make RSSI alone deficient for estimating the distance between people across scenarios [2]. In cases where people are close in metal enclosures such as public transportation, research has also shown that there is little or weak correlation between phone distance and measured RSSI [3] and thus close contact cannot be correctly detected by the current Google/Apple Bluetooth contact tracing systems.

Because of the above issues, it is important to investigate alternative sensing approaches that are suitable for wide-scale, privacy-respecting sensing on smartphones while compensating the above problems of the BLE RSSI approach.

### 1.1. Paper Contributions

In this paper we investigate the use of ambient sound intensity fingerprints to support social distancing monitoring and contact tracing with mobile devices. The specific contributions of the paper are:

1. Our proposed concept uses privacy-preserving ambient sound intensity fingerprints for social distancing monitoring. The idea is to leverage the fact that ambient sound received by a mobile device is a superposition of sounds from sources at many different locations in the environment. Such a superposition is determined by the relative position of those sources with respect to the receiver. Thus two receivers at the same location are likely to see a very similar superposition and receivers further away will see more different superpositions. To preserve privacy, we work with temporal patterns of intensity profiles at a single fixed frequency which does not allow the reconstruction of sensitive information such as spoken words or speaker identity.
2. We have designed and implemented a signal processing chain for translating differences in the fingerprint received into proximity indication.
3. We demonstrate how this proximity information can be used to classify distance between users carrying a mobile device with respect to the 1.5 m social distancing threshold. This includes sound-only classification as well as fusion with a classical BLE RSSI approach.
4. We validate our method on a real-life data set from different locations in a city (supermarket, hardware store, train station, office building, mall, etc.), showing an accuracy of up to 80% for sound and up to 86% combined (Bluetooth alone 77%).
5. We present different evaluation modes to demonstrate the strengths and limitations of each of the modalities and to help users understand where and how sound can help. This includes showing in an additional small lab experiment that sound can detect physical barriers that prevent infections even when two users are physically close (e.g., glass or office walls).

### 1.2. Related Work

(A) Social Distancing

Social distancing is one of the most effective physical approaches to slow the spread of the pandemic besides mask-wearing [4] and lock-down measures [5]. The WHO declared a social distancing of at least 1 m among patients and healthcare workers. Generally, a distance of 1.5 m to 2 m is regarded as a reliable distance to decrease the infection risk [6]. However, this is an extra burden for people when the social distancing rule needs to be applied, especially for hospital workers who need to focus on their duties. In these cases it is helpful to support social distancing and contract tracing by technically aided approaches [7]. There are a series of sensing modalities with distance or proximity monitoring as a basic functionality, such as ultrasound [8,9,10], Ultra Wide Band (UWB) [11,12,13], magnetic field [14,15], electric field [16], visual-based sensing [17] etc. Ultrasound and UWB are wave-based active approaches that would be able to deliver distance information with a very high accuracy but suffer from the large amount of deployment work, and are only suitable for indoor distance monitoring. An artificial magnetic field system could be worn on the body and supply cm-level distance accuracy. Unfortunately, an ease-of-use and comfortable wearable device is still not at hand. Another novel solution is based on the electric field emitted from the human body, namely the body capacitance. The static electric field of an object will be deformed once an intruder occurs within a range. With a simple, low-cost, low power consumption smart band-like device, the proximity of an intruder could be captured. However, it only works reliably when the object is in a static state. The visual solution is a mature one for social distancing alerting, benefiting from developments using deep learning for computer vision. However, it causes privacy concerns, since it reveals more information than needed for social distancing. In practice, the technique-aided social distancing solution should be primarily accurate in distance estimation and secondly accessible for most people. Thus we have focused on sensing units that are available on most smartphones, trying to show their ability in social distancing.

(B) Bluetooth-Based Position Estimation

The Bluetooth Received Signal Strength Indicator (BT-RSSI)-based approach is naturally a popular one considering the pervasiveness of the Bluetooth-embedded everyday personal digital assistant, the smartphone. The RSSI signal of Bluetooth has long been explored for distance estimation applications like indoor positioning [18,19], proximity sensing [20,21], etc. The current pandemic boosted the introduction of BT-RSSI sensing for solving the contact tracing problem. However, previous related studies showed that this signal is quite weak (easily being affected by antenna, surroundings, obstacles, etc.) in distance estimation when a certain accuracy was required. To address this issue, researchers have explored advanced signal processing methods. Vivek et al. [22] proposed a method by using temporal features of BT-RSSI to boost their distance estimation model’s accuracy. Similar work was explored by Arun et al. [23]. However, the above-described approaches with the input of just Bluetooth data are still likely to be too poor [24,25]. Even Google/Apple’s epidemiological assessment model on the BT-RSSI signal(Google/Apple’s Exposure Notification [26]) tends more to assess the exposure risk level instead of inferring more concrete information on social distancing. Douglas et al. [3,27] have worked on evaluating the measurement of Google/Apple exposure notification API for proximity detection in a commuter bus and a light-rail tram. Unfortunately, the real-life experiment concluded that in the tram there is little correlation between the Bluetooth signal strength received and the distance between handsets, and in the bus that there is only a weak dependence of detection rate on distance. Considering the experiments were performed in metal-rich environments with limited space, that can cause more complex radio reflection and refraction than other environments, their result is reasonable.

Other works have tried to improve RSSI-based recognition by including other modalities present in the smartphone. For example, Shankar et al. [28] achieved promising results by combining Bluetooth with acceleration and gyroscope, among others, but their models do not generalize across datasets, probably because deep learning models can easily overfit the overall characteristics of any environment, specially with ambiguous modalities such as Bluetooth-Based position estimation. Other analyses such as [29] have pointed out the limitations of RSSI alone when not taking into account device placement and sampling interval.

(C) Sound Based Proximity Sensing

As described above, a social distancing solution solely depending on BT-RSSI might result in a high potential for false positives. Thus we are considering including other sensing modalities. Sound has also been used in this way in an infrastructure-type deployment (e.g., using pre-installed microphones [30] or as a self-calibrating distributed platform [31]). However, since people are inherently mobile, the sensing modalities used to detect social distancing should share that characteristic. Thus the ideal aiding approach should be yielded from devices used everyday close to people. Here sound signal shows excellent potential, since it can be captured by the microphones on all smartphones. Previous work on sound captured by microphones on smartphones are mainly focused on indoor location [32,33], proximity detection [34,35], or other environmental assessment applications [36,37], with the sound sources either from fine-tuned sound tags or from the surroundings. Under ideal conditions, it has been shown that sound can be used to accurately measure distance using smartphones alone. Peng et al. [38] demonstrate a high accuracy ranging system (but requiring line of sight). In a given unobstructed and low-noise environment, even 3D localization is possible, as shown by [39]. In real-world, noisy situations, however, these approaches are not feasible. Wirz et al. [40] have shown that a fuzzy proximity detection approach is feasible using sound fingerprinting, but accuracy is low (46% only for distance estimation). It is therefore reasonable to aim for a multimodal approach that is still capable of being executed on smartphones.

To the best of our knowledge, no research work has been done that aims to improve the accuracy of BT-RSSI-based social distancing by considering the environmental sound recorded by the phones in a privacy-respecting way.

## 2. Approach

### 2.1. General Idea

Our environment is full of ambient sounds: people talking, ventilation/air conditioning, the sound of cutlery in restaurants, various machine noises, cars in the streets, birds singing, wind and so on. At any given point in time, a mobile device’s microphone will receive a superposition of the corresponding sound waves. How the signals from different sources superimpose depends on their relative location with respect to the receiver. With the speed of sound being around 340 m/s, even a few meters of difference in terms of the relative distance between the sound source and the receiver will influence the details of the superposition. So will any sound absorbing/reflecting surfaces between each of the sources and the receiver. As a consequence, two receivers that are physically close to each other are likely to see a similar superposition pattern, while ones that are further away will register more differences in the received signals. In particular, unlike with Bluetooth Low Energy (BLE), two devices separated by a physical barrier (that also prevents infection) such as a glass sheet or a thin wall will likely see very different signals.

When applying the above idea, two things must be considered. The first is privacy. Continuously recording sound from which private conversations may be inferred in a social setting is in general inappropriate and, in many countries, legally problematic. Secondly, people may be storing their devices in very different locations: from a pocket that has the microphone fully exposed to being buried deep in a bag. This means that comparing the absolute intensity of the received signal at any given point in time is not a good strategy. While sound is originally sampled at a usual frequency of 48 k, to address privacy concerns, it is processed on the mobile device and only saved in its processed form. In detail, the original sound signal on the device is buffered into jumping windows of 5 ms length, then the mean of these windows is taken and saved as a 200 Hz feature vector. Note that this does not suffer from artifacts due to low sampling frequency, as the initial signal still had the regular 48 k often used for sound recording. The frequency of 200 Hz was chosen as the highest frequency available that still supported real-time processing on all devices. A human inspection of the signal made sure that it did not result in any identifiable speech. To overcome the varying intensity problem, we compute time series similarity measures for appropriate intensity normalized time windows, thus focusing our system on the shape of intensity variations that result from the different ways in which signals from different distances and directions are superimposed at different locations.

Our aim is to apply the above approach to alleviate the shortcomings of Bluetooth-only methods by fusing sound information and BLE RSSI signals to infer user proximity more reliably. In the rest of this section we describe the sound processing approach in more detail, present the BLE RSSI analysis method that we employed (we rely on standard methods as BLE is not the focus of this work) and outline the fusion and classification strategy applied to both signals.

### 2.2. Sound Processing

Initially, we hoped that under ideal circumstances it might be possible to infer at least vague distances from the sound time of flight by matching segments of different users and calculating the time shift in between. Real-life signals, however, were too noisy to achieve that goal.

Thus, a first approach that was less prone to noise and more robust in calculating similarity between people was developed using Dynamic Time Warping (DTW) as a similarity measure. However, this approach also fell short of our expectations, as DTW was prone to random bursts of high distance estimates that degraded the overall performance.

**Figure 1:**
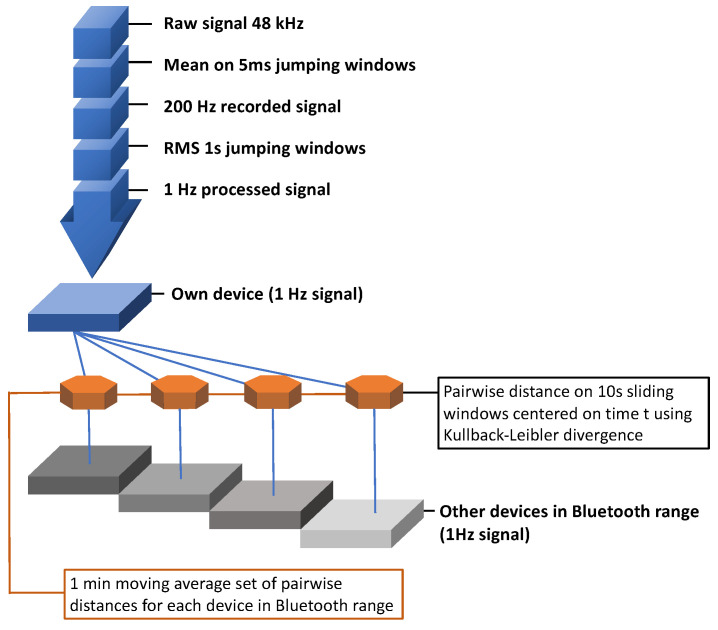
Sound-processing algorithm.

As a consequence, we searched for a better similarity measure, settling on the Kullback-Leibler divergence, which was used in the final algorithm given below (also shown in Figure 1). Please note that the first two processing steps in this figure are described above and happen on device, before sound is stored.

1. From the 200 Hz amplitude feature vector recorded on the device, calculate the root mean square (RMS) on 1 s jumping windows. Note: storing this as a float value needs 4 bytes per second, for a total of 240 bytes per minute. This is a consideration since for the following steps, we assume that people share the information recorded in step 1) with fellow tracing app users.
2. Assuming we call our own signal S and the set of n other people who are at least in Bluetooth visible range $O_{1}$ to $O_{n}$: divide the signal into windows centered on the current data point extending 5 s into the past and future between every pair (S, $O_{i}$). Note: this leaves the temporal granularity unchanged at 1 s. For each of those windows, create a histogram distribution of the sound amplitudes (empirically, 20 bins has proven to be both robust and detailed enough). For each pair of histograms between S and each other device, calculate the Kullback–Leibler divergence as a measure of similarity. The Kullback–Leibler divergence is defined as $D_{\mathrm{KL}}\left(P\|Q\right)=\sum_{x\in X}P\left(x\right)\log\left(\frac{P(x)}{Q(x)}\right)$ Since this is not commutative for P and Q, we calculate the average of both orders of inputs, i.e., $(D_{\mathrm{KL}}\left(P\|Q\right)+D_{\mathrm{KL}}\left(Q\|P\right))/2$
3. Finally, calculate a moving average of 1 min.

**Figure 2:**
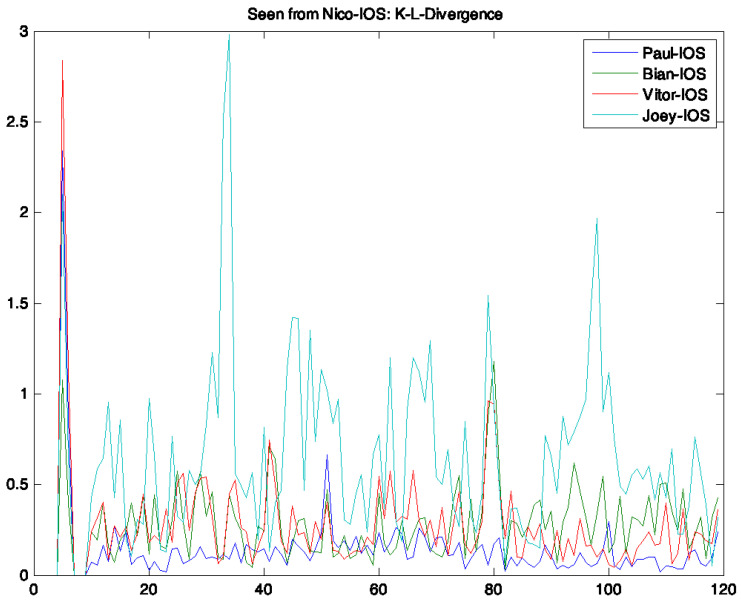
Example of Kullback–Leiber divergence (KLD) distance from one device to several others. The *y*-axis shows the calculated KLD, the *x*-axis time in 1 min intervals.

Figure 2 gives an example of various Kullback–Leiber divergence time series between pairs of devices. It is apparent that the measure does discriminate between different device pairings.

During initial trials, it also became apparent that some kind of filtering for sound ’quality’ was required. Two main circumstances may occur in a real-life setting:

1. Devices may not record any sound whatsoever. This may happen due to technical reasons, incoming calls, etc.
2. Devices may not record a significant level of ambient sound. One may argue that periods of silence interrupted by interesting intervals of sound are actually the norm in many environments.

To provide this filter information to later classification steps, every window that had no sound information at all was marked as such (we chose a label of −2 for that since amplitude information is never negative). As a clarification, −2 really means no sound at all, e.g., because users received a phone call (which is not recorded) or turned off their device to pass a checkpoint. Every window that lacked meaningful information (i.e., where total amplitude was below a threshold determined as the value that marked the 10th percentile of all amplitudes observed) was marked with −1. On choosing the 10th percentile as a threshold: the aim was to preserve a meaningful amount of sound while discarding the least informative (i.e., the quietest) parts. Setting the threshold value to the 10th percentile accomplishes that. Any sound that exceeds the background (i.e., the range from 10.01% to 100% of recorded amplitudes) is allowed to register, but any sound below that is filtered out. If the window was not marked with −2 or −1, it was considered as a “good” sound window and marked with a 1.

An important aspect of any sensing system is the influence of noise. A key advantage of our approach is that the signal it uses is essentially “noise”. We do not generate or try to detect any particular sound; we merely compare the ambient sound (=noise) received by the devices of two users. “Noise” in the sense of confusing or misleading information would only consist of sounds that are generated extremely close (much closer then the 1.5 m social distance, in general centimeters) to one of the devices. Such sounds generate large intensity differences independently of the distance between the respective users. In our experiments we have not observed this problem. Should it occur, absolute intensity or the magnitude of the intensity difference could be used to filter such sounds out.

### 2.3. Bluetooth Processing

The use of RSSI as a means of distance estimation is a well studied topic (see Related Work) and the general processing chain is well understood. Since the focus of this work is not on RSSI proximity detection as such, but on the added benefit of sound analysis, we rely on standard methods for RSSI processing.

**Figure 3:**
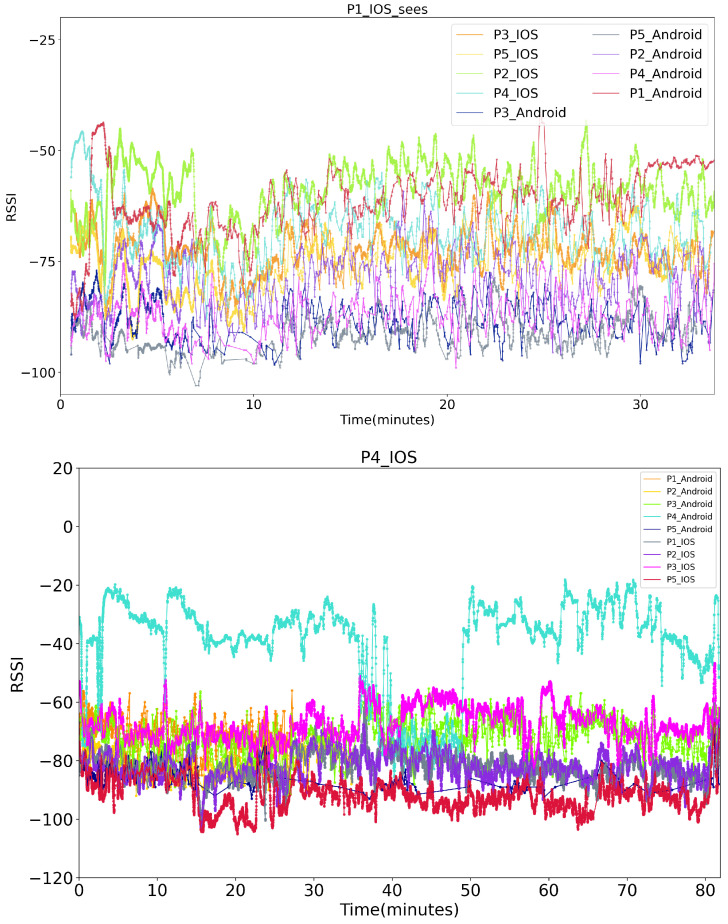
(**Top**): An example of recorded RSSI (dBm) raw value by P1’s iPhone in our five-volunteers experiment. (**Bottom**): More stable RSSI (dBm) values seen by P4’s iPhone after compensation and smoothing.

Bluetooth RSSI is a weak signal easily affected by many factors such as the antenna’s orientation and obstacles between the transmitter and receiver. This is illustrated in Figure 3. It shows an example of recorded RSSI by one of the iPhones during the experiment (see section Evaluation) where two subjects, P1 and P2, were walking at largely constant close distance (below the social distancing 2 m limit) and subject P5 was constantly several meters away. It can be seen that while on average the different relative distances are reflected in the signal, there are also very significant temporal variations that do not correspond to any real change in the distance between the subjects.

To address such variations we applied two pre-processing steps to the raw data.

1. Transmission power compensation
  A smartphone’s Bluetooth transmission power setting plays a considerable role in RSSI-based distance estimation [41]. Mimonah et al. [42] validated that by considering transmission power when estimating the distance, reduced the mean errors by over 35% in varied distance estimating models, and increased the proximity classification accuracy by over 70%. RSSI data is essentially describing the relationship between transmitted power and received power of an RF signal. This relationship is stated in the following Equation (1) [43]:
  (1) $$P_{r}=P_{t}{\left(\frac{1}{d}\right)}^{3}$$
  where $P_{r}$ is the receiving power, $P_{t}$ is the transmission power and *d* is the distance between them. By performing the logarithm of both sides 10 times, we get the dBm expression:
  (2) $$P_{r}\left(dBm\right)=P_{t}\left(dBm\right)-10log\left(d\right)$$
  Equation (2) shows a linear relationship of the transmission power and the received power for a certain distance. In our app, the initial transmission power was included in each received data package and was recorded together with the signal strength data. Thus the first step we applied to the recorded Bluetooth RSSI signal was the compensation. By simply mining the Tx power of each phone, we kept the Tx power consistency of every smartphone as if they had the same value with zero dBm.
2. Moving-average smoothing
  Regarding the instability of the RSSI data caused by the antenna’s surrounding and environmental complexity, we then performed a moving-average smoothing process on the compensated RSSI data. The smoothing was based on a ten second sliding window with every new RSSI value as one forward step, so that the delay caused by the smoothing can be neglected. Figure 3 depicts the RSSI values recorded by P4’s iPhone after steps of transmission power compensation and smoothing, which shows a more stable RSSI path of tested phones.

**Figure 4:**
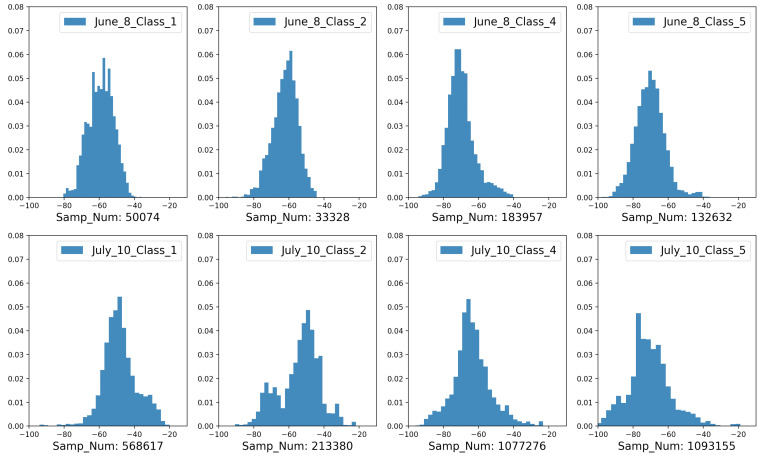
Distribution of pre-processed BT-RSSI values regarding four classes (Class 1: below 1 m, Class 2: 1.5–2.0 m, Class 4: around 3 m, class 5: beyond 5 m).

Figure 4 depicts the BT-RSSI distribution of the four classes collected in 2 days after compensation and smoothing.

### 2.4. Signal Fusion and Mapping onto Proximity Classes

Next the sound similarity estimation and pre-processed RSSI signals must be translated into proximity information. For social distancing analysis and contact tracing, we are not interested in exact numerical distance estimates on a fine-grained time scale (e.g., person A being 1.25 m from person B at time t1 and 1.35 m 1 s later). Thus, for example the risk computation in the Apple iOS and Google Android contact tracing workflow is based on an opportunistic scanning strategy that only promises a minimum periodic sampling every 5 min [44], where Tx corrected RSSI Bluetooth data are collected and aggregated into 8 pre-defined discrete ranges (bins). The bin where the user stayed most of his time is then used as the probability score of the user having been in “dangerous” proximity to another person. This information is then combined with other risk criteria (days since symptoms, etc.) and used to estimate the infection risks.

As a consequence, we focus our analysis on distinguishing between being below the social distancing limit (“Very Close”, below ≈1.5 m, P1 and P2 in the datasets, and above it (“Further”, above ≈1.5 m, all other pairs in the datasets). In order to see how the system behaves with more granularity, we also experimented with three classes: The same “Very Close” for people not social distancing, but now we separate people who are between 1.5 and 5 m (“Mid or Social Distancing” (P3 and P4 in Figure 9) to those that are even further away (“Very Far”). We initially apply the analysis to 5 second jumping windows assigning each one to one of the above classes. We then investigate various majority decision strategies for different longer time frames, resembling more the several min long iOS/Android analysis.

Every 5 s window always has one feature related to Bluetooth, which is a minimum RSSI value the source device can see of the target after our Bluetooth pre-processing procedure. Sound features will only be available in windows that have good sound, that is, when both phones have marked their windows with a 1 in our sound filtering process. In that case the window will have one extra value representing its sound similarity, which comes directly from our sound data pre-processing as it also provides one value per 5 s jumping window. We explored different classification strategies in order to evaluate our privacy preserving sound and Bluetooth approach:

1. Our baseline, i.e., classification using only the Bluetooth data.
2. Classification using only the sound data. This can only be done when both phones have good sound, so this restricts the number of windows that can be classified. When comparing this approach with the Bluetooth one, we will compare predictions on only those windows.
3. The combination of both, which is our proposed approach. It consists of training one classifier with only Bluetooth features that will be applied if there is no sound feature for a window and another classifier that was trained using sound and Bluetooth that will predict labels for windows that also have sound distances.

As we are interested in detecting whether or not people have stayed close for longer periods of time, each window will take into account the previous 4 min (48 windows) by determining labels through soft voting. As we hypothesize that sound can improve social distancing classification, we will weight windows that also use sound features 20 times more than others in cases where we are mixing features (case 3). For this window weighting we experimented with values of 1, 7 and 20, with increasing (but diminishing) returns in classification improvement. Notice that with a weight of 20 the voting between windows is almost dominated by predictions that use sound and Bluetooth together, but we will not discard/disregard other windows as sound features require both devices to be measuring reliably the ambient sound, which may happen sparsely in some environments.

For all classifiers, we used the CART decision tree [45] implementation from the sklearn library [46]. Since our datasets are highly unbalanced, we weight instances inversely proportionally to their frequency, i.e., instances of class $c_{i}$ have weight $\frac{\sum_{c}N\left(c\right)}{N(c_{i})}$ where N(c) is the number of samples for *c* in the training set. While we explored other classifiers and even grid search on the tree’s parameters by k-fold validation on the training set, the best configuration in all cases was a decision tree of maximum depth 4, minimum sample split of 5 instances and entropy as split criterion. We selected this approach as it was less prone to over-fitting, as the distribution of Bluetooth data is influenced by many factors including scenario, phone placement and orientation, etc.

## 3. Experiments

We evaluated our approach in two steps. We first performed a controlled experiment in our lab environment to verify the hypothesis that sound fingerprint analysis is better in detecting thin physical barriers often used to reduce the infection risk rather than BLE RSSI. We then performed a large scale experiment in a real life environment by walking for several hours (on four different days) with a group of people spaced at different distances through various settings in the city and the university campus recording sound fingerprints and RSSI values with iOS and Android smartphones.

### 3.1. Controlled Lab Scenario

As discussed in the introduction a major drawback of BLE RSSI proximity sensing (and most other RF sensing methods) for social distancing monitoring is the fact that thin physical barriers, that are very effective at preventing infections, have nearly no effect on the signal. Thus, two people sitting on different sides of a office wall will likely be registered as possible contacts, although in reality the wall perfectly blocks infection.

**Figure 5:**
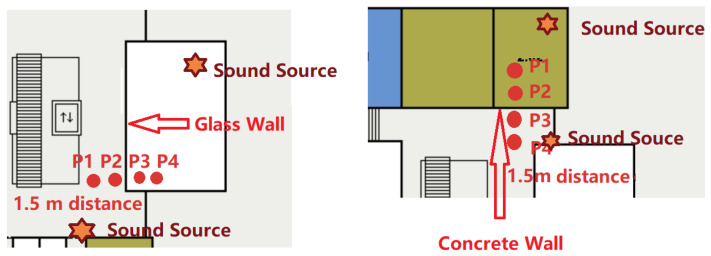
A preliminary sound and Bluetooth test with a concrete wall and glass wall between the smartphones(P1, P2, P3, and P4 were the four phones statically on tables of the same height. We brought two sound sources inside/outside the rooms to simulate environmental sound information. The sound was from a YouTube video playing mixed sounds from a supermarket. The phones were placed in line 1.5 m apart.

Figure 5 illustrates two experiments we have performed to verify that adding the proposed sound fingerprinting method alleviates that problem. We have recorded ambient sound for 4 devices in a line with just under 1.5 m (the social distancing boundary) in between with two (P1 and P2) being in a corridor and two (P3 and P4) being in an office (with a wall in between) or in a seminar room (with a glass separation to the corridor). Ideally P1/P2 and P3/P4 should be detected as being potentially dangerous contacts while all the others (in particular P2/P3) should be registered as being outside the danger zone (as there is a barrier in between).

**Figure 6:**
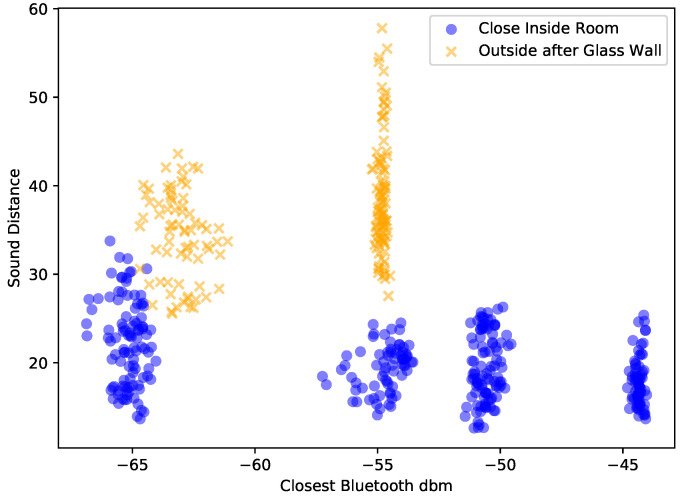
Distribution of features for all our 5 s windows that had acceptable sound for our wall experiment.

We have collected data for around 17 min to get enough data points, calculated the Bluetooth and sound features and plotted them for the phones that should register as possible contacts and the ones that should not. The data are shown in Figure 6. Since it was so clear, there was no need to train a classifier to validate the hypothesis. We can see that Bluetooth alone could not differentiate between being close on one side of the wall and being close but with a wall between. On the other hand, our sound similarity provided a better separation, especially if it was combined with the Bluetooth feature.

**Figure 7:**
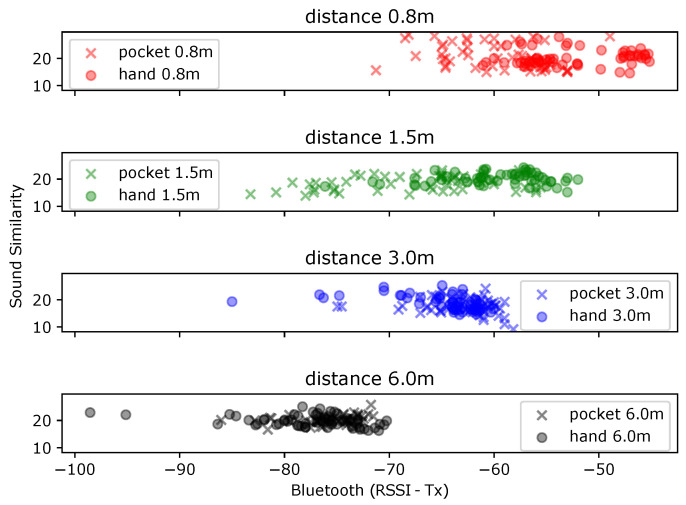
Distribution of features for our pocket versus hand experiment. We can see that there is overlap in Bluetooth readings for different distances and that phone placement can influence them too. Sound similarity, on the other hand, is more consistent with a difference in range only when very close (0.8 m).

Another relevant factor is placement of the phone. More specifically, how phone placement affects Bluetooth and sound readings. We analyzed this by performing another controlled experiment where a subject was holding one phone in their hand and another in the pocket. We then placed a single sound source closer to a third phone and varied the subject’s distance to it, recording for each distance 5 min of Bluetooth readings and sound similarity. The data can be seen in Figure 7, where we can see that in this controlled scenario Bluetooth was affected by placement while sound similarity was more consistent.

### 3.2. Real-Life “City Scale” Data Recording

**Figure 8:**
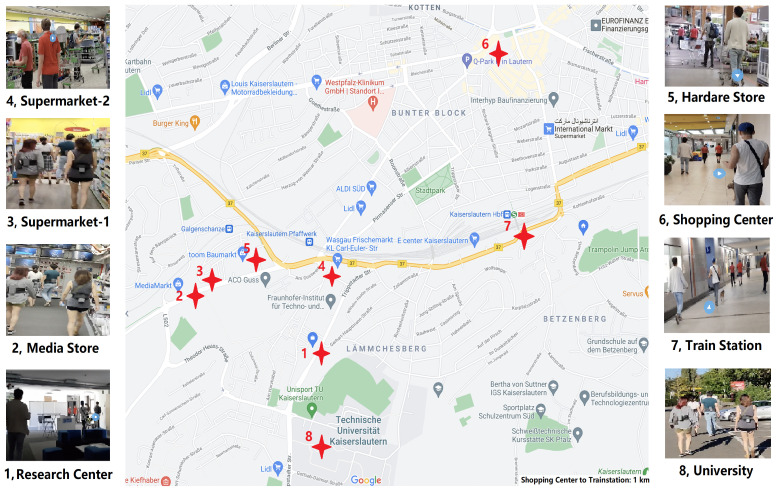
Places where data were collected.

**Figure 9:**
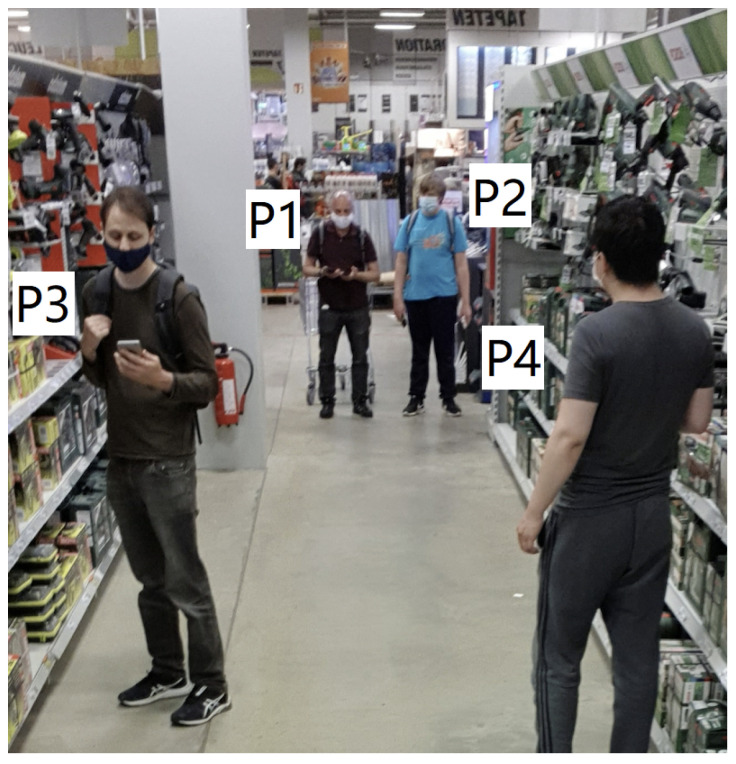
Collecting data in a DIY supermarket. In this case P1 and P2 are very close, while P3 and P4 are at social distancing. We call the distance between both groups (for example between P1 and P3) medium distance. Not seen in this picture is P5, who is even further away in another aisle. We call the distance to the subjects here Very Far.

To record both BT and sound data with a smartphone, we developed customized apps for both iOS and Android platforms. Timestamp was attached to each received package for synchronization. Each of our smartphones broadcasts a legible ID (to circumvent the Bluetooth MAC anonymization strategy [47]) while at the same time scanning for packages from the other smartphones (based on their known IDs). Figure 8 depicts two days of experiments with five volunteers carrying two smartphones, one in each hand- The places we visited included the university campus, a research center building (went through several floors, corridors, stairs, social areas), a media store with plenty of electrical devices, a large DIY store with plenty of construction products made of metal, wood, liquid, concrete, etc., two supermarkets for daily necessities, a large shopping center with various stores, restaurants, etc., and a train station. Overall, we used data recorded from four days for our study. During the recording activities, the five volunteers were arranged into three groups, as Figure 9 presented in detail, as the first group, P1 and P2 were always side by side to keep a very close distance, P3 and P4 with a distance of 1.5 m as the second group maintaining a social distance. The two groups held a range of 3–4 m as a medium distance. P5 was far beyond the two groups with at least a distance of 5 m, regarded as a class of very far. All volunteers wore medical masks during the data recording. Altogether we used four days of recorded data (over three hours) to explore the contribution of environmental sound information to BT-RSSI-based social distancing monitoring.

**Figure 10:**
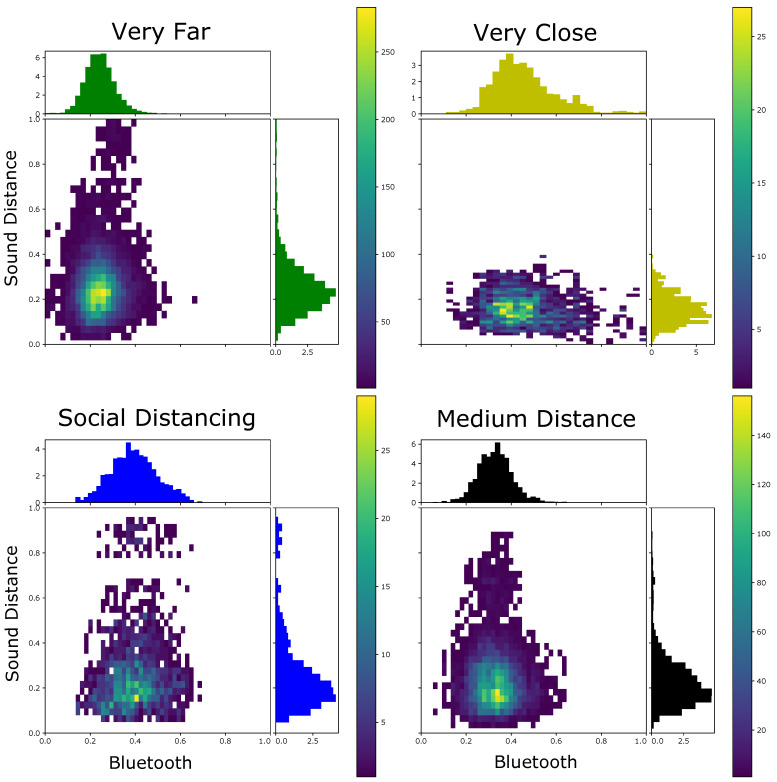
Distribution of features for all our 5 s windows that had acceptable sound. The sound feature represents the dissimilarity of the sound between the two subjects and was calculated using the one minute averaging, while the Bluetooth one represents the minimum pre-processed Bluetooth signal recorded by the subject of the other. Participants who were very close experience similar sound features, and thus sound similarity can solve some of the ambiguity present using only Bluetooth signals.

A more complete description of our recorded data is displayed in Table 1. There we present the places that were visited on each recording day and also the availability of both Bluetooth and sound across windows. Any contact tracing approach that relies on Bluetooth assumes that this signal is available. For all days we have more than 79% of the windows with Bluetooth readings, meaning that in theory this is mostly achievable (even if not desirable due to higher battery consumption). Another important question for our method is the availability of useful sound information. As expected, this varied over the days, from almost 19% to a little over 48%. This poses a limit on the performance of sound alone in practice, but shows that there is potential for significant improvement if it is useful. This usefulness is also a consideration as we now have data in real life scenarios with much more variability than our previous controlled tests. In Figure 10 we show the distribution of features for all our 5 s windows that had good sound on all our recorded days. There we can see that different distances generated distinct Bluetooth distributions, but there wasa a lot of overlap between them, even if we wanted to classify only between socially distancing or not. More interestingly, people who were very close presented more similar sound distances. Intuitively, this should make the inclusion of sound beneficial as we can use it to confirm that people were not socially distancing in cases where the Bluetooth readings were ambiguous. On the other hand, dissimilar sound alone could not predict that people were far apart, as they can present very dissimilar sound even when social distancing.

## 4. Results

### 4.1. Effect of Physical Barrier in Lab Setup

The result of the “physical barrier” lab experiment described in Section 3.1 and sketched in Figure 5 is shown in Figure 6. The blue dots show signals from pairs of devices that were close to each other while being in the same room (the same side of the barrier). The yellow crosses are from devices that were also close to each other, but with a barrier (wall, glass) in between.

It can clearly be seen that with Bluetooth alone (*x*-axis) the physical barrier cannot be detected as the blue dots and yellow crosses completely overlap within the first two horizontal clusters. At the same time, they are nearly perfectly separated in the vertical dimension which represents sound.

### 4.2. Social Distance Detection in Real Life Environments

#### 4.2.1. Evaluation Methodology

In the evaluation procedure we consider the following aspects:

**Sensing modality.**  We considered the classification using Bluetooth alone, sound alone and the fusion of both modalities.
**Window vs. Event-based evaluation.**  The most basic test for classification performance is to look at each individual window and compare the results achieved with the three possible sensing modes above. When doing this we must however consider that sound is not present in all windows (as at times the environment may be silent). We thus do the comparison only on windows that have valid sound. For the purpose of social distancing monitoring and contact tracing 5 s temporal resolution is clearly not needed. It thus makes sense to consider aggregated predictions in larger windows. We found 4 min (=48 windows) to be a good trade-off between temporal resolution and accuracy. Again, the fact that not all windows had sound needed to be taken into account (see below). Finally since typically 15 min periods are used in most contact tracing approaches, we will also investigate the performance of the system with respect to “events” of two people being close to each other for a period of at least 15 min.
  In summary we will present the following evaluation modes:
  1. Evaluating both modalities and their fusion, using only windows where there is a valid sound signal. This is the “baseline” for a comparison between all modalities on specific data points (Section 4.2.2).
  2. Aggregation over groups of 48 windows of 5 s windows with valid sound. Windows that do not have valid sound are ignored. This is the most effective way to use sound. However it overestimates the usefulness of sound information by ignoring the fact that, as opposed to Bluetooth, sound does not provide a prediction for all windows (Section 4.2.3).
  3. Aggregation over 4 min intervals always taking into account all consecutive windows, no matter if they have valid sound data or not. Thus we include sound information wherever it is available and make a pure Bluetooth-based decision wherever there is no sound. This accounts for the fact, that no matter how useful the sound information is, it only helps with a certain fraction of windows (Section 4.2.4).
  4. Performance on aggregated decisions in 15 min windows to account for typical time scales in most existing contact tracing apps. This includes plain aggregation on 15 min and an analysis of all pairs of subjects to determine if those two subjects spent at least one 15 min time interval together below the 1.5 m social distancing limit (Section 4.2.5).
**Number of classes.**  The key performance metric is the ability to distinguish between being below and above the social distancing boundary of 1.5 m. This is our main evaluation metric. However, in order to understand the sensitivity of the sound information better we will also present results for a three class problem as described in Section 2.4.
**Choice of training/testing days.**  As described in Section Figure 8 of the four days on which we recorded data, each two were in different types of environments (first: inside shops, offices, street, second: train station, mall, university canteen). As, especially for Bluetooth, signal attenuation is highly environment dependent we use the different types of data to investigate the sensitivity of the performance of each of the modalities to the similarity between the training and the testing environments.

The discussion below is structured along the “Windows vs. Event” aspect with all the other aspects being varied for each of the cases 1–4 as described above.

#### 4.2.2. Evaluation on 5 s Windows with Valid Sound Signal

**Figure 11:**
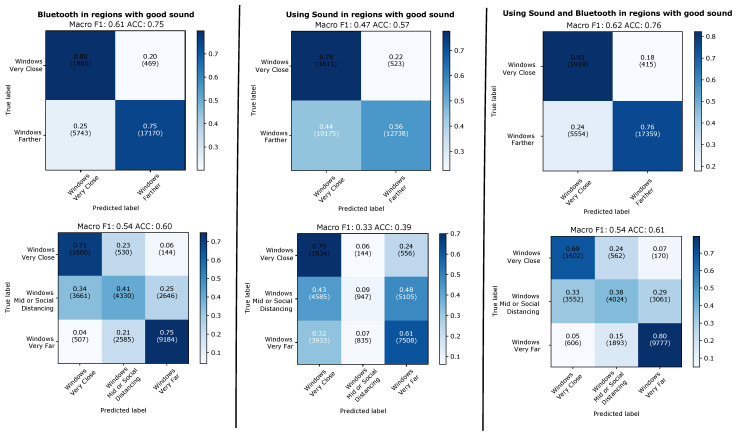
Leaving-one-day-out. Results predicting individual 5 s windows with good sound.

**Figure 12:**
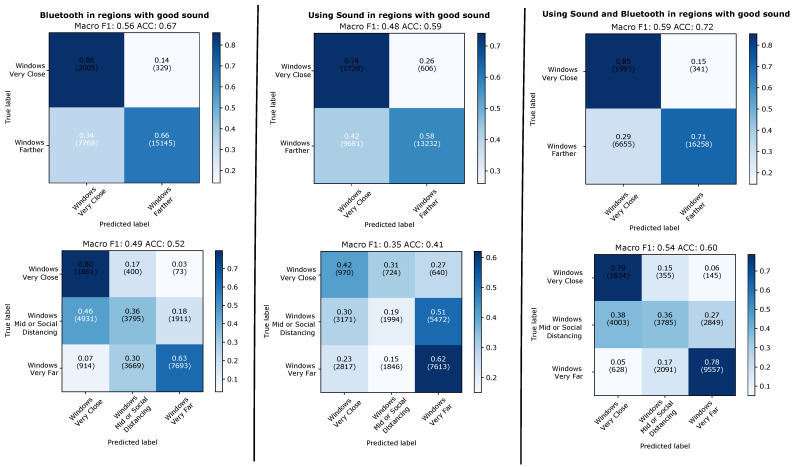
Evaluation across types of days. Results for evaluation when predicting individual 5 s windows with good sound.

The results of the 5 s window evaluation are shown in Figure 11 for the leave-one-day-out case and in Figure 12 for the case on training and testing on two different types of days. On individual 5 s window level sound alone is significantly worse then Bluetooth alone (between 14 and 21 % on the F1 score, between 8 and 21% on the accuracy). However, even on individual window levels adding sound to Bluetooth nearly always improves performance (between 1 and 5 % on the F1 score, between 1 and 8 % on the accuracy, with one case in both accuracy and F1 being 0% difference). Sound tends to perform better on the two class problem and slightly worse on the three class problem. This is not surprising since, as shown in Figure 10, sound is particularly good at modelling the “very close” class. Furthermore the difference between Bluetooth and sound is smallest (and the benefit of adding sound to Bluetooth largest with the improvement from 49 to 54% on F1 and from 52 to 60% on accuracy for the three class case) when the training and the test set come from different types of environments. This is also in line with our expectations, as RF signal attenuation tends to be much more prone to environmental factors than the type of differential sound fingerprint signal that we consider.

#### 4.2.3. Aggregation over 48 Windows of 5 s with Valid Sound Signal

**Figure 13:**
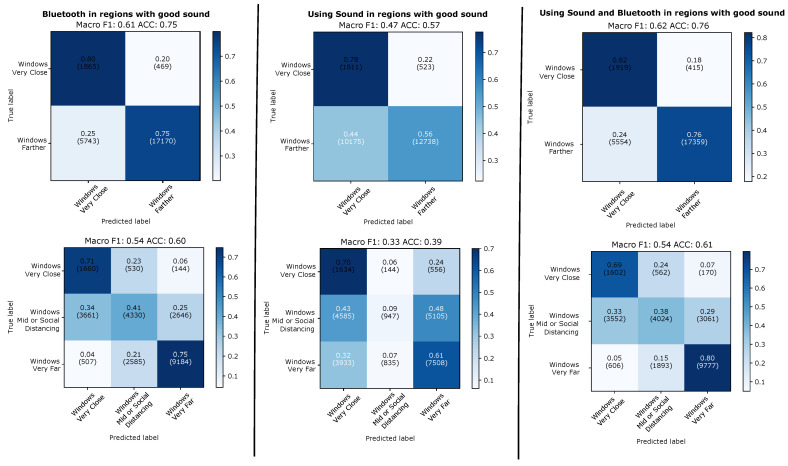
Leaving-one-day-out. Results predicting individual 5 s windows with good sound.

**Figure 14:**
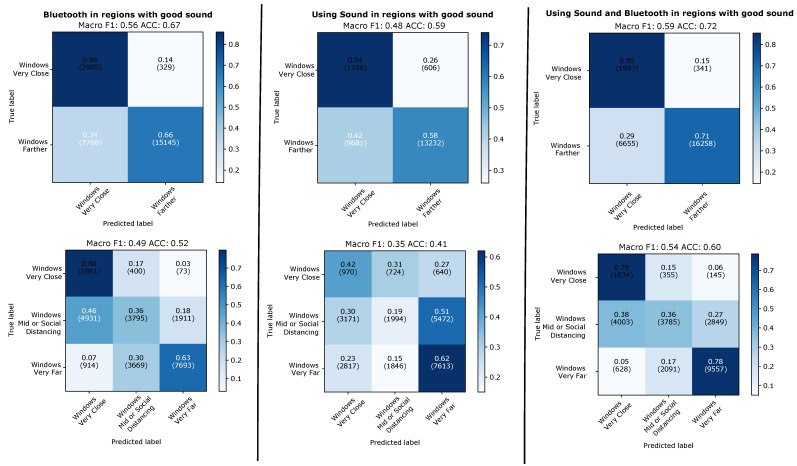
Evaluation across types of days. Results for evaluation when predicting individual 5 s windows with good sound.

The results of 48 window aggregation can be seen in Figure 13 for the leave-one-day-out and in Figure 14 for training and testing on different types of days. It can be seen that the aggregation significantly improves the performance over the individual windows analysis. It can also be seen that the improvement is much more pronounced for sound and fusion of sound and Bluetooth (between around 10 and 20%) and much smaller for Bluetooth (between 5 and around 10% maximum). For the two class problem sound outperforms Bluetooth with a best score of F1/accuracy of 63/80% that rises to 72/86% for the best case of the fusion of the two modalities (when for the same case Bluetooth alone reaches just 63/77%).

Given the relatively poor performance of sound in a window by window level (as compared to Bluetooth) it may at first seem surprising why the aggregation leads to such a large improvement. However, if we consider the types of errors occurring in the sound signal, then this is to be expected. Many errors occur in situations where the distribution of ambient sound sources (or the way the phone is kept) means that although the system has sound signals that it considers valid, it is not possible to extract proximity from it. In general this is not a result of “fast variations” like a person walking by and blocking the Bluetooth signal. Instead this tends to be a longer term (minutes) phenomena. This means that many of the window level errors are clustered together, when such phenomena occur. In other words many wrongly labeled individual windows contribute to relatively few errors on an aggregated level.

It is also important to note that taking only windows with valid sound for the aggregation means that the 48 windows we aggregate for each data point do not always represent 4 min. This is because quite often in between two windows with valid sound signals there are going to be one or more windows with where no valid sound was recorded. For the usefulness of the approach for contact tracing it is essential to know how much time is needed to collect the 48 windows relates to the 15 time span typically used in contact tracing systems. This is visualized in the bottom part of Figure 13. It can be seen that in the vast majority of cases we need between 5 and 10 min to collected the 48 windows, with effectively no instances significantly beyond 15 min.

#### 4.2.4. Aggregation over 4 min on all Windows

**Figure 15:**
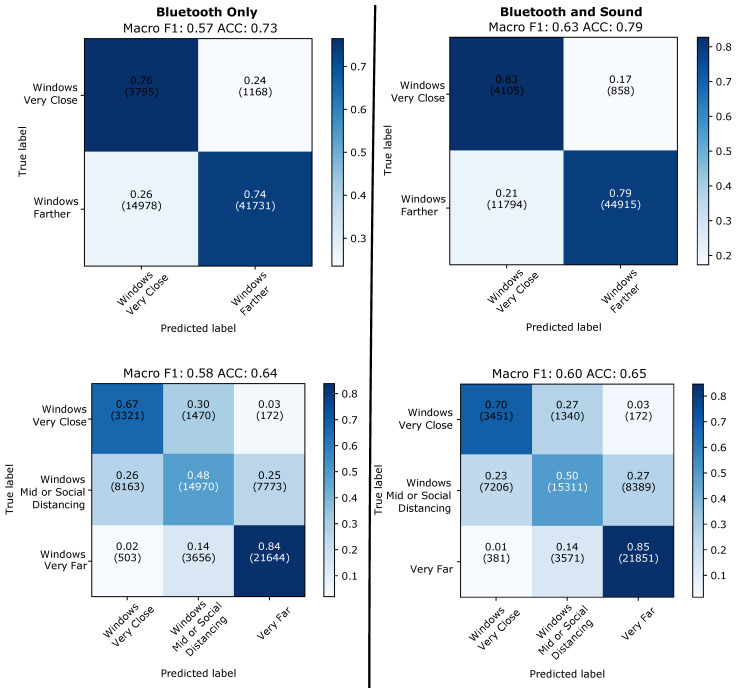
Leaving-one-day-out. Results of aggregation over 4 min taking into account all windows (with and without sound).

Beyond the question of how long it typically takes to collect the 48 windows, we need to understand the impact of the fact that at times there may be significant periods with no valid sound at all. As explained in Table 1 valid sound is present only in 20 and 50% of the recording time as opposed to over 90% on all days for Bluetooth. Thus we consider the aggregation over 48 consecutive windows (=4 min) no matter if they contain valid sound or not. To this end we train two models: One trained on all data that has sound and Bluetooth features and another trained only on Bluetooth. The choice of model to use when predicting a window will then depend on the features available at run-time. As in our classification scheme 5 s jumping windows are voting on the current label, it makes sense for windows with good sound to have greater weight in the aggregation if including sound can discriminate better between the classes. This also increases the possible influence of windows with good sound, as they are underrepresented (see Table 1). In our experiments we tried weights of 1, 7 and 20, with 20 giving the best results. Leaving-one-day-out, this approach produces the results displayed in Figure 15. We have a 6% improvement in macro F1 for binary classes and 2% for the case with three classes. Moreover, we can see 7% of all “very close” windows correctly classified in the binary case and overall improvement in all classes, showing that it is realistic to expect sound to improve classification overall even if it is not always present.

**Figure 16:**
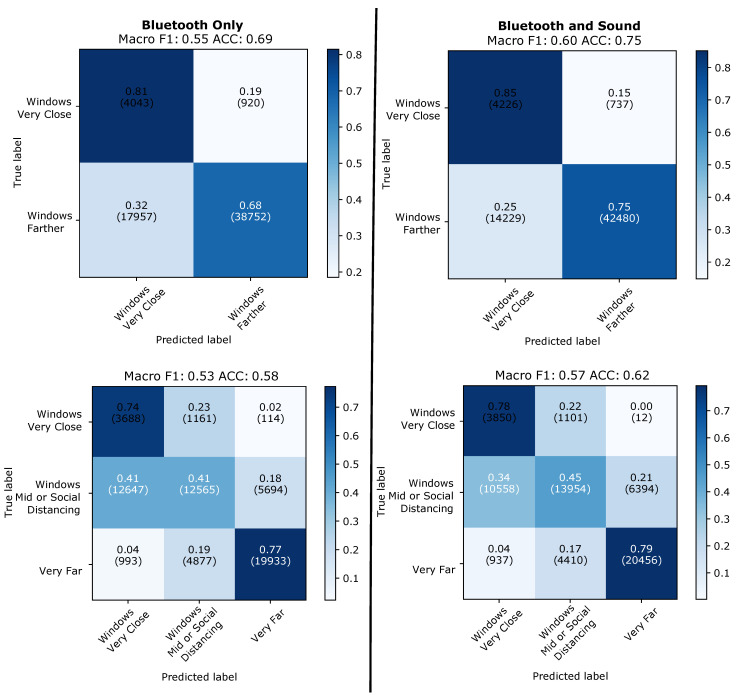
Train one type test another. Results for all windows.

If we apply the same procedure leaving one type of day out (Figure 16) we can see the same patterns, with Bluetooth plus sound improving classification for all classes and providing results closer to the “leave-one-day-out” counterpart compared to the Bluetooth only approach.

#### 4.2.5. “Event” Based Evaluation on 15 min Windows

When doing contact tracing we are interested in whether or not two persons spend more than 15 min in close proximity. It is by automatically capturing those interactions that a contact tracing system can inform people to quarantine and get tested, thus breaking the infection chain of the virus. As a final evaluation step we thus examine the 15 min windows performance in two ways: looking at each 15 min window separately and checking how many pairs of users who have spent at least 15 min below the social distancing threshold are recognized on each test day.

**Figure 17:**
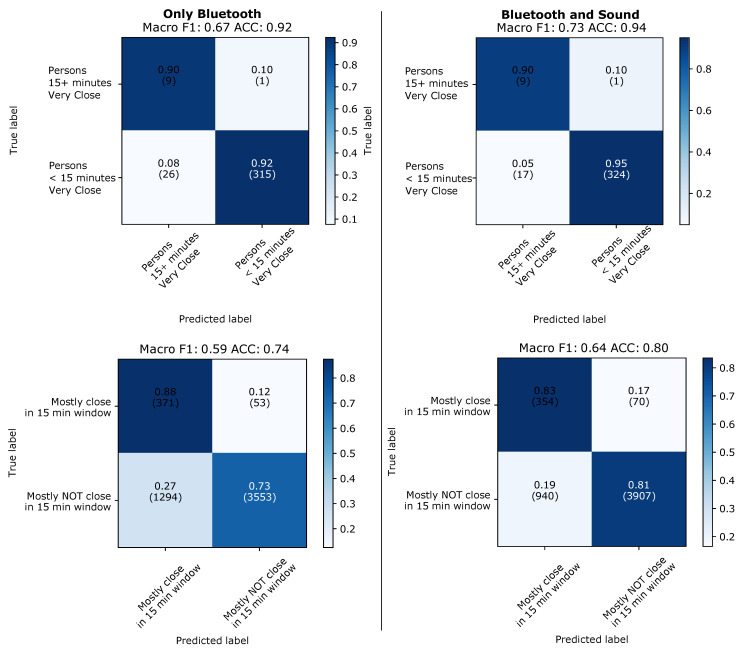
Leaving-one-day-out. Comparing people that can be detected being close for 15 min or more using different features.

Those two metrics can be calculated by aggregating our window predictions for the binary case. Doing that for leave-one-day-out results we get the results in Figure 17. There we can see that the inclusion of sound helps us avoid false positives in our contact tracing system, as with only Bluetooth 26 persons would be incorrectly notified versus only 17 for Bluetooth and sound. As both methods have 9 true positive notifications, the 6% improvement in macro F1 comes at no penalty. If we look at the results for the 15 min windows, we can see 8% less false negatives, which contributes to a 5% increase in macro F1, but some of the true positive windows that Bluetooth captures are lost when we also use sound. On the other hand, the overall change is positive, especially given the order of magnitude difference between class sizes and the fact that those lost windows do not really change who would be contacted.

**Figure 18:**
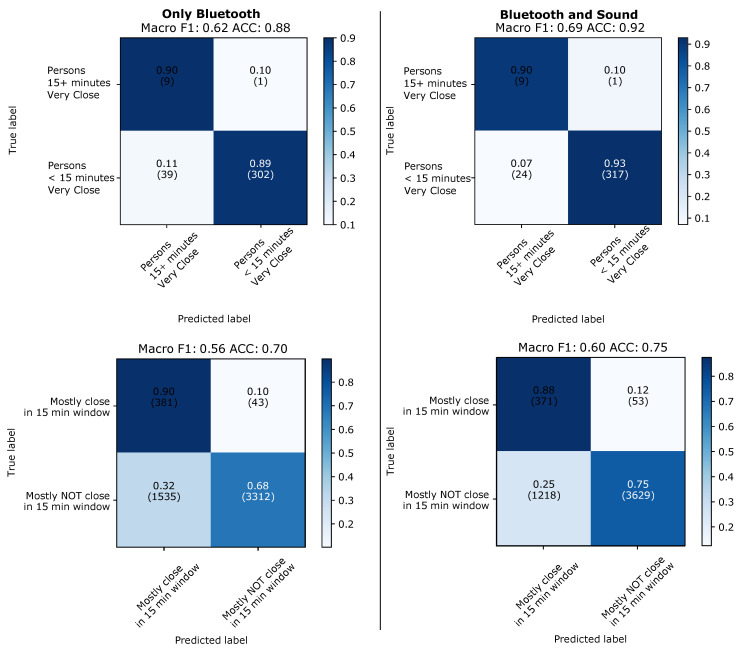
Train one place, test on the other. Comparing people that can be detected being close 15 min or more using different features.

If we apply the same analysis for leaving out one type of day, we can see similar results (Figure 18). Again Bluetooth alone overestimates the number of contacts generating more false positives. As the overall window classification tends to be better when also including sound, we hypothesize that even Bluetooth’s edge in the 15 min case would disappear given more data.

## 5. Conclusions

Overall our work clearly demonstrates the usefulness of ambient sound fingerprints for anonymous social distancing and contact tracing, in particular as an addition to Bluetooth sensing. Key takeaways are

1. As shown in Section 4.1 and Figure 6 sound fingerprints can reliably detect physical barriers which negate proximity as a potential infection risk. By contrast using Bluetooth alone two people sitting on opposite sides of an office wall could be detected as a potential infectious contact.
2. As outlined in Section 4.2.3 and shown in Figure 13 and Figure 14 focusing on windows that have good sound signal and aggregating over 48 such windows allows the system to recognize being below the social distancing range with an accuracy of over 80%, improving the purely Bluetooth based recognition.
3. Bluetooth and sound complement each other in many ways beyond the ability of sound to detect physical barriers. Most importantly, the majority of factors that lead to errors are very different due to the different physical nature of the signals. Bluetooth tends to have problems with environments with a lot of metallic structures and many people moving, which are both not an issue for sound. On the other hand, fairly empty environments (except for a few people who are potentially infectious contacts) tend to be quiet and thus challenging for sound analysis, but are very well suited for Bluetooth. Finally, while there are some conditions in which sound does not work at all, it is much less sensitive than Bluetooth to the specific conditions in which the system is trained being identical to the conditions where it is deployed.

It is important to note that the above has been demonstrated, not with full sound recording but with a constraint intensity pattern analysis avoiding any privacy issues at the cost of utilizing only a small amount of the information contained in the sound signal. In future work, it should be investigated how a more comprehensive analysis of the sound signal can be done on user devices by extracting relevant features in real time without actually recording any privacy-sensitive information. In this context, the real time mobile implementation is another research challenge. Furthermore, it is interesting how the two modalities compare in more complex environments such as, e.g., a factory floor, which is known to be very challenging to RF localization in general while at the same time containing a lot of clear ambient sound.

There are some other key limitations of the method presented:

1. While we have conducted an initial small experiment to get an indication of the influence of different phone storage locations, further more detailed studies are clearly needed. This includes the problem of noises that are generated very close to the microphone, which can be expected to occur, e.g., when the phone’s microphone rubs against a tight pocket.
2. Our sound analysis approach performs poorly for the middle class in the scenario with three classes (around 1.5m as opposed to very close and very far). The physical principle that we use does not in any way imply such a limitations, although it is clear that the two class problem is harder. For practical reasons we have optimized our system to distinguish very far and very close. In future work a more fine grained resolution needs to be specifically addressed.
3. The current method of all or nothing distinction between valid and invalid sound signal in each window clearly has limitations. So does the notion of a static weight for the sound signal. It is likely a key constraint on the performance of the current system. In the long term an adaptive dynamic approach is needed, which reflects the level of uncertainty in both the Bluetooth and sound signal and weights the inputs accordingly.

## Figures and Tables

**Table 1:** Description of the days when we recorded data. Time in each day is the experiment duration, but instances are generated between phones being held by different people, so the number of 5 s windows exceeds the experiment duration given that experiments included five subjects, each carrying two phones (1 iOS and 1 Android). Percentages of availability are calculated using those windows between users. The days are divided into two types (A and B) based on the locations visited.

Day	Type	Places Visited	Windows	Windows	Duration
		(Bluetooth)	(Good Sound)	(Minutes)	
1	A	train station, university	92.7%	48.2%	61
2	A	train station, university	92.4%	47.1%	86
3	B	research center, media store, hardware store, supermarket	79.67%	18.9%	30
4	B	hardware store, shopping center	94.2%	37.2%	16

## Data Availability

Data used in this study can be obtained from the authors. Please send an email request.
